# The shadow of trauma: impaired mentalization in clinical populations – a systematic review

**DOI:** 10.1017/S0033291725100822

**Published:** 2025-07-08

**Authors:** Marianna Gorgellino, Geetanjali Kumar, Yusra Parkar, Ana Catalan, Natalia Fares-Otero, Martin Debbané, Marco Armando, Luis Alameda

**Affiliations:** 1 Treatment and Early Intervention in Psychosis Program (TIPP), Department of Psychiatry, Lausanne University Hospital (CHUV), Lausanne, Switzerland; 2 Department of Psychosis Studies, Institute of Psychiatry, Psychology, and Neuroscience, King’s College London, London, UK; 3 Department of Psychiatry, Basurto University Hospital, Bilbao, Spain; 4 Biocruces Bizkaia Health Research Institute, Barakaldo, Spain; 5 Department of Neurosciences, University of the Basque Country (UPV/EHU), Leioa, Spain; 6 Centro de Investigación Biomédica en Red de Salud Mental (CIBERSAM), Instituto de Salud Carlos III, Madrid, Spain; 7 Department of Psychiatry and Psychology, Bipolar and Depressive Disorders Unit, Institute of Neurosciences (UBNeuro), Hospital Clinic of Barcelona, IDIBAPS, Barcelona, Catalonia, Spain; 8 Faculty of Psychology and Educational Sciences, University of Geneva, Geneva, Switzerland; 9 Research Department of Clinical, Educational, and Health Psychology, University College London, London, UK; 10 Division of Child and Adolescent Psychiatry, Department of Psychiatry, Lausanne University Hospital (CHUV), Lausanne, Switzerland; 11 National Psychosis Unit, South London and Maudsley Foundation TRUST, National Health Services, London, UK

**Keywords:** childhood maltreatment, mental disorders, metacognition, neglect, psychosis, social cognition, theory of mind

## Abstract

**Background:**

Mentalizing defines the set of social cognitive imaginative activities that enable interpretation of behaviors as arising from intentional mental states. Mentalization impairments have been related to childhood trauma (CT) and are widely present in people suffering from mental disorders. Nevertheless, the link between CT exposure, mentalization abilities, and related psychopathology remains unclear. This study aims to systematically review the evidence in this domain.

**Methods:**

A Preferred Reporting Items for Systematic Reviews and Meta-Analysis (PRISMA)-compliant systematic review of literature published until December 2022 was conducted through an Ovid search (Medline, Embase, and PsycINFO). The review was registered in the Prospective Register of Systematic Reviews (PROSPERO) (CRD42023455602).

**Results:**

Twenty-nine studies were included in the qualitative synthesis. Twenty studies (69%) showed a significant negative correlation between CT and mentalization. There was solid evidence for this association in patients with psychotic disorders, as almost half the studies focused on this population. The few studies focusing on unipolar depression, personality disorders, and opioid addiction also reported a negative impact of CT on mentalization. In contrast, evidence for post-traumatic stress disorder was inconsistent, and no evidence was found for bipolar disorder. When stratifying for subtypes of CT, there was solid evidence that neglect (physical and emotional) decreased mentalization capacity, while abuse (physical, emotional, or sexual) was not associated with mentalization impairments.

**Conclusions:**

Although causality cannot be established, there was substantial evidence that CT negatively affects mentalization across various psychiatric disorders, particularly psychotic disorders. These findings highlight the potential of targeting mentalization impairments in prevention and treatment strategies aiming to reduce the incidence and the social functioning burden of mental illness.

## Introduction

Mentalization defines the set of social cognitive imaginative activities that enable the interpretation of behaviors as arising from intentional mental states. This capacity enables individuals to imagine and understand their own mental states, as well as those of others, including thoughts, feelings, intentions, and desires. As such, mentalization is crucial for effective social interactions and the ability to adapt in complex social contexts (Fonagy, Gergely, Jurist, & Target, 2002). The concept of mentalization is closely linked to metacognition, Theory of Mind (ToM), reflective functioning, cognitive empathy, and social cognition (Fonagy, Luyten, & Strathearn, 2011; Lysaker, Gagen, Moritz, & Schweitzer, 2018). While metacognition emphasizes more on self-reflection and regulating one’s own cognitive processes, mentalization, cognitive empathy, and Theory of Mind – a key component of social cognition – primarily focus on understanding others. Reflective functioning, a core aspect of mentalization, centers on the comprehension of attachment-related experiences and emotions within relational contexts. Despite their distinct emphases, these interconnected concepts collectively underscore the complexity of human social-cognitive abilities, essential in enabling self-awareness, complex social interactions, and navigation of both personal and others’ mental worlds.

Historically, research on mentalization deficits has focused primarily on borderline personality disorder and psychotic disorders, contributing significantly to understanding these conditions. In individuals with borderline personality disorder, mentalization theory has played a central role in explaining emotional dysregulation and interpersonal dysfunction, with early attachment disruptions and trauma hypothesized to impair the development of reflective functioning, a conceptual framework that led to the development of Mentalization-Based Treatment (MBT), an evidence-based intervention for this disorder (Bateman & Fonagy, 2008). In the context of psychosis, mentalization deficits have been investigated as a part of a broader disruption in social cognition, with relevant implications for daily functioning, contributing to social withdrawal and isolation (Lysaker et al., 2005).

Although these two populations have received the most attention, growing evidence suggests that impaired mentalization may also play a role in other psychiatric conditions, providing the basis for a transdiagnostic perspective.

Recent studies showed that mentalization deficits are more prevalent among individuals with psychiatric disorders than in the general population (Nazarov et al., 2014; Healey, Bartholomeusz, & Penn, 2016; Trauelsen et al., 2016; Nemeth et al., 2018). Notably, these deficits do not seem to result from the disorder itself, as they are unaffected by symptom severity or disease stage, and may be evident as early as the prodromal phases or even prior to the onset of mental illness (Addington et al., 2008; Green et al., 2008; Green et al., 2012). Consistent with these findings, there is preliminary evidence that deficits in mentalization may constitute a transdiagnostic risk factor closely associated with broader psychopathology, potentially underlying vulnerability across various psychiatric disorders (Bateman, 2012; Luyten, Campbell, Allison, & Fonagy, 2020).

Mentalization deficits not only signal vulnerability to various psychiatric disorders but also play a critical role in shaping social and functional outcomes across these conditions (Sharp & Venta, 2013). In this sense, several studies have shown that mentalization capacity plays a crucial role in social functioning across various psychiatric disorders; individuals with mentalization impairments often experience significant challenges in social interactions and overall functioning (Couture, Penn, & Roberts, 2006; Green et al., 2008; Bell, Tsang, Greig, & Bryson, 2009). In conditions like psychosis, mentalization – alongside other aspects of social cognition – emerges as a key determinant of functional outcomes, suggesting that improving mentalization deficits could meaningfully enhance general functionality (Fares-Otero, Alameda et al., 2023).

It is well established that traumatic events during childhood, typically classified as physical and emotional neglect and physical, sexual, and emotional abuse, represent a well-known and highly prevalent risk factor for the onset of mental illness (McKay et al., 2021). Indeed, evidence indicates that the prevalence of childhood trauma in psychiatric populations ranges from 70% to 85% (Battle et al., 2004; Larsson et al., 2013; Kessler et al., 2017), while in the general population, it is approximately 30% (Larsson et al., 2013; Whitten, Tzoumakis, Green, & Dean, 2024). Although childhood trauma is consistently associated with various mental health conditions, the mechanisms underlying their link are not fully understood yet (Alameda et al., 2020), and the role of mentalization in the interplay between childhood trauma and psychopathology requires further exploration.

In recent years, research has indeed identified trauma, in the form of abuse and neglect, as a significant risk factor for impaired social cognition, including mentalizing abilities (Ensink et al., 2017; Rokita, Dauvermann, & Donohoe, 2018) in both clinical and nonclinical populations. It is suggested that traumatic events occurring during early development interfere with the formation of mentalizing abilities during childhood and adolescence (Fonagy & Luyten, 2009; Ensink et al., 2017; Rodriguez et al., 2021; Martin-Gagnon, Normandin, Fonagy, & Ensink, 2023). Moreover, mentalization deficits identified in populations exposed to traumatic events during childhood have been linked both to trauma by abuse (Cicchetti et al., 2003; Pears & Fisher, 2005; Ensink et al., 2015) and to trauma by neglect (Shipman & Zeman, 1999; Edwards, Shipman, & Brown, 2005).

Despite evidence linking mentalization abilities to trauma exposure, only two studies cover this area (Rokita et al., 2018; Yang & Huang, 2024). Rokita’s systematic review concluded an association between the social environment experienced during childhood, including traumatic experiences, and poorer social performance as an adult, including social cognition. However, specific insights on the link between childhood trauma and mentalization were not provided, and the review’s search was conducted in May 2018, with considerable research emerging since then (Kincaid et al., 2018; Quide et al., 2018; Rnic et al., 2018; Weijers et al., 2018; Mansueto et al., 2019; Simon et al., 2019; Trauelsen et al., 2019; Guhn et al., 2020; Li, Carracher, & Bird, 2020; Eidenmueller et al., 2021; Rokita et al., 2021; Vaskinn, Melle, Aas, & Berg, 2021; Branas et al., 2022). Yang’s meta-analysis evidenced a negative correlation between childhood trauma and mentalization capacity. However, this work only included five studies conducted in clinical samples, mainly focusing on the general population. While similar reviews have examined social cognition in psychotic disorders (Fares-Otero, Alameda et al., 2023), and in mood disorders (Fares-Otero, De Prisco et al., 2023), none, to our knowledge, has taken a transdiagnostic approach with a focus on mentalization. This review aims to address that gap by concentrating on mentalization, now recognized as a promising target for psychological treatment. Disentangling the relationships between childhood trauma, mentalization, and subsequent psychopathology is essential for developing new prevention and treatment strategies to reduce the incidence and social functioning burden of mental illness.

## Methodology

This systematic review was registered with the International Prospective Register of Systematic Reviews (PROSPERO; registration number: CRD42023455602). The review was reported according to the updated Preferred Reporting Items for Systematic Reviews and Meta-Analysis (PRISMA) guidelines (Page et al., 2021) (see Table S1 in the supplement) and the Enhancing the Quality and Transparency of Health Research (EQUATOR) reporting guidelines (Altman et al., 2008).

### Search strategy

A systematic search was conducted in Ovid (Medline, Embase, PsycINFO), covering studies from database inception to December 2022. The search strategy included keywords related to (1) psychiatric populations, (2) childhood trauma (CT), and (3) mentalization domains. The Boolean operator ‘AND’ was used (see search strategy and terms inserted in the supplement). After a consensus between different mentalization-based therapy experts (MA and MD), we decided to include in our search all terms relating to mentalization, namely ‘Theory of Mind’, ‘metacognition’, ‘reflective functioning’, ‘social cognition’, and ‘mindfulness’. This was necessary as these concepts are overlapping, sometimes used interchangeably, and many clinical trials use the same assessment tools to measure different constructs. Given the variability in terminology across studies, we opted for a broad search strategy to minimize the risk of overlooking potentially relevant papers.

Filters were applied to remove duplicate entries, research that was not conducted on human samples, and papers that were not published in the English language. After retrieving the articles, two researchers (GK and YP) independently screened the titles and abstracts of identified articles for relevancy. There was a 92% agreement consistency between both researchers; any discrepancies were resolved by two senior researchers (LA and MA). Subsequently, two researchers (GK and YP) independently conducted the full-text screening to assess eligibility; any discrepancies were resolved by a third researcher (MG) at a group meeting. Additionally, a manual literature search was performed to identify further eligible articles from the reference lists of previously identified reviews and included studies. This included: (i) screening the references of included studies, and (ii) screening the references of articles cited by the most relevant reviews on this topic (Rokita et al., 2018; Rodriguez et al., 2021; Fares-Otero, De Prisco et al., 2023; Fares-Otero, Alameda et al., 2023; Yang & Huang, 2024).

### Inclusion and exclusion criteria

In relation to the PECO(S) (Participants, Exposition, Comparators, Outcomes, Study Design) framework (Morgan, Whaley, Thayer, & Schünemann, 2018), studies required (1) (P) for clinical studies of any psychiatric diagnosis based upon validated diagnostic manuals and scales, which includes the Statistical Manual for Mental Disorders (DSM-IV and V) and the International Classification of Diseases (ICD-10 and 11), or psychiatric symptoms within the general population (GP); (2) (E) examined the presence of CT, defined as having occurred before the age of 18 and measured as composite CT or specific CT subtypes (sexual, physical and emotional abuse, physical and emotional neglect); (3) (C) compared with individuals without CT; (4) (O) assessed for mentalization outcomes (mentalization, Theory of Mind, metacognition, reflective functioning, social cognition, cognitive empathy, and mindfulness) with validated instruments and its relationship with CT; (5) reported quantitative results; (6) reported original results from a peer-reviewed journal. Predictors, Outcomes, and Table S3 in the supplement include the definitions and operationalization outcomes for each domain. Studies were excluded if (1) they were reviews, clinical cases, conference proceedings, study protocols, dissertations, or gray literature; (2) included a sample of participants over the age of 65 years.

### Data extraction and quality assessment

Researchers (GK and YP) extracted data from all included studies into a Microsoft Excel database. The data was then cross-checked by a third researcher (MG) to ensure high data extraction quality. The descriptive variables extracted consisted of the following: (1) first author and year of publication and country; (2) sample size, ICD-defined/ DSM-defined or general population (GP), mean age (with range), and percentage of female; (3) CT measure used and subtype of trauma reported; (4) mentalization domain and outcome measure; (5) covariates with the analysis; (6) results in the association between CT and mentalization. Two independent reviewers (GK and YP) utilized the modified version Newcastle–Ottawa Scale (NOS) (Wells et al., 2014) to assess for quality and risk of bias. This was cross-checked by a third reviewer (LA). Detailed information regarding the quality assessment process is presented in Table S4 of the supplementary material.

## Results

### Summary of search results

**Figure 1.**
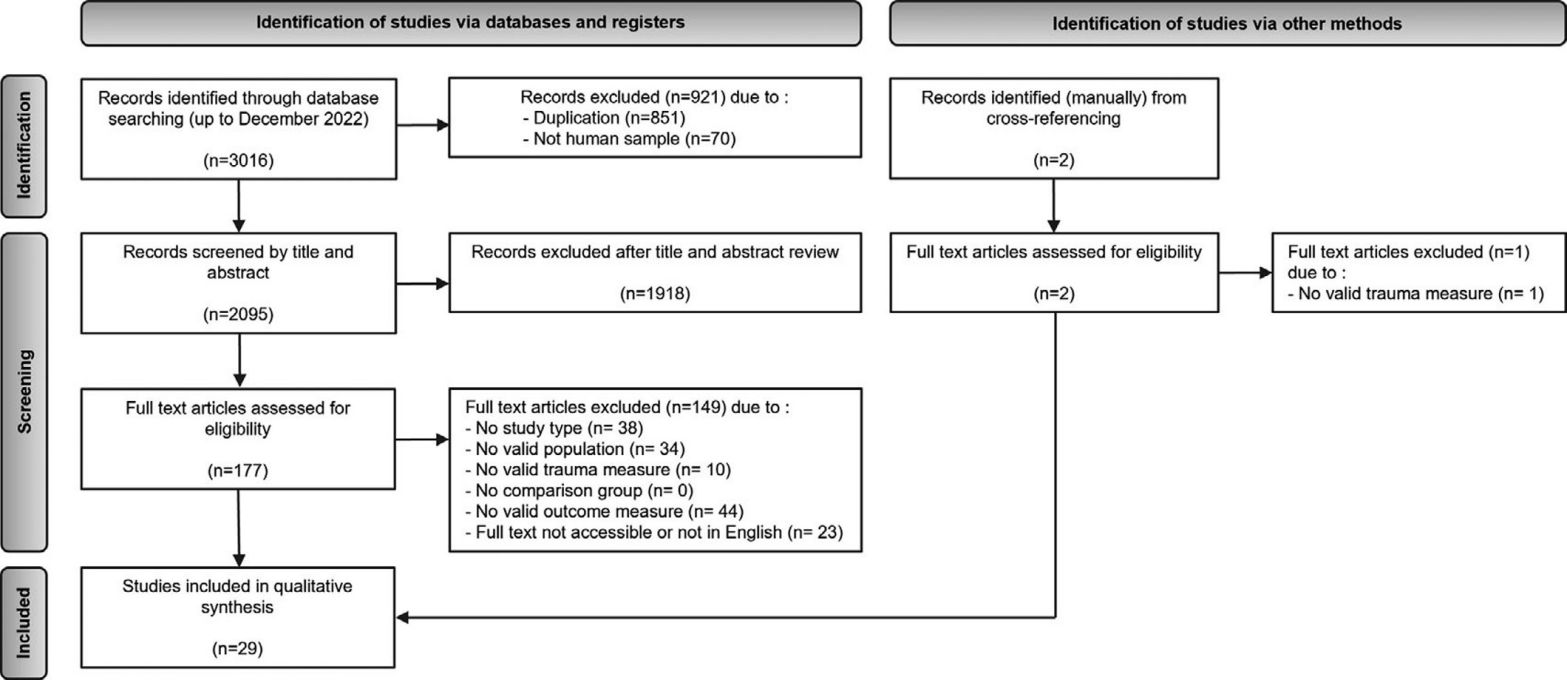
PRISMA 2020 flowchart of studies selected for systematic review.

Of 3018 eligible articles, 179 were full-text screened and 29 were included in the qualitative synthesis. The details of the selection process have been visually displayed through the PRISMA flow diagram in Figure 1. The final selection and a summary of the findings are presented in Table 1.

**Table 1.** Characteristics of the studies included in the review

Author (Year), Country	Sample,Mean Age, % of Female	Childhood Trauma measures	Mentalization domain and measures	Main findings	NOS score (Max = 8)
Addington et al. (2019) Canada	CHR = 40 17, 53%	Semi-structured interview Composite	Metacognition MAS-A	Metacognition scores were unrelated to CT	5
Andreou et al. (2015) Germany	BPD = 44 29, 86% SCZ = 36 32, 44% HC = 38 33, 56%	CTQ Composite	Social Cognition MASC	BDP and SCZ exhibited different social cognition impairment patterns (respectively overmentalization and undermentalization) but CT was not a significant mediator of these impairments	6
Aydin et al. (2016) Turkey	SCZ = 35 30, 37% HC = 35 31, 60%	CTQ Composite & Subtypes (PA, EA, SA, PN, EN)	Metacognition MAS-A	Lower metacognitive abilities related to EA but not to any other subtypes of CT	6
Belvederi Murri et al. (2017) Italy	All diagnosis = 83 17, 69% HC = 145 17, 68%	CTQ Composite	Mentalization MZQ	Mentalization deficits were associated with the severity of depressive symptoms and the severity of CT; 40% of the effect of CT on depressive symptoms was mediated by mentalization deficits	6
Branas et al. (2022) Spain	Psychotic disorder = 62 31, 47%	Semi-structured interview Composite & Subtypes (PA, EA, SA)	Social Cognition, ToM HT	No significant association between CT and ToM performance was found	6
Brune et al. (2016) Germany	BPD = 30 26, 100% HC = 30 25, 100%	CTQ Composite & Subtypes (PA, EA, SA, PN, EN)	Mentalization Novel Cartoon Task	Decreased mentalization abilities were associated with PA, PN and EN but not with EA nor SA; PN and EN predicted respectively 33% and 25% of the variance of mentalization	6
Chiesa and Fonagy (2014) United Kingdom	Personality disorders = 122 33, 72% HC = 122 33, 69%	Cassel Baseline Questionnaire Composite & Subtypes (PA, SA, PN, EN)	Mentalization, Reflective functioning RFS	All subtypes of trauma significantly decreased the reflective functioning abilities; reflective functioning was confirmed as a significant mediator between CT and personality disorders	7
Eidenmueller et al. (2021) Germany	SUD = 66 43, 32% HC = 66 41, 46%	CTQ Composite & Subtypes (PA, EA, SA, PN, EN)	ToM MASC	ToM impairments were associated with PA and PN but not with EA, SA, EN	5
Garcia et al. (2016) Spain	Psychotic disorder = 79 26, 39% HC = 58 24, 48%	CTQ Composite & Subtypes (PA, EA, SA, PN, EN)	Social Cognition MSCEIT-ME	CT was associated with poorer social cognition; when stratifying for subtypes of CT, this association remained significant only for PN and EN	6
Guhn et al. (2020) Germany	MDD = 68 41, 63% HC = 34 41, 59%	CTQ Composite	Cognitive empathy, Mentalization IRI, MET	CT was not associated with cognitive empathy scores	7
Jimenez et al. (2017) Spain	BD = 113 49, 60%	CTQ Composite & Subtypes (PA, EA, SA, PN, EN)	Social Cognition MSCEIT-ME	CT was not associated with social cognition performance	7
Kilian et al. (2018) South Africa	FEP = 56 24, 25% HC = 52 25, 33%	CTQ Composite & Subtypes (PA, EA, SA, PN, EN)	Social Cognition MSCEIT-ME	PN and EN were significantly associated with poorer social cognition performance, while PA, EA and SA were not	5
Kincaid et al. (2018) Northern Ireland	SCZ = 66 45, 21%	TEC Composite & Subtypes (PA, EA, SA, EN, life threat, harassment)	ToM HT	EN, specifically occurring at age 0–6, predicted ToM impairments in SCZ population; EN accounted for 8.7% of the variance of ToM; none of the other CT subtypes was associated with ToM impairments	6
Li et al. (2020) Scotland	GP-depressive symptoms = 205 28, 81%	ACE Subtypes (PA, SA) CEAS Subtypes (EA) CECA Subtypes (PN, EN)	Mentalization RFQ	EA, PN and EN correlated with mentalization impairments and the level of depressive symptoms, while PA and SA did not; both hypomentalizing and hypermentalizing mediated the association between EA and depressive symptoms (mediation analysis were not performed for other subtypes of CT)	6
Lysaker et al. (2011) USA	SCZ and Schizoaffective disorder = 101 46, 15%	TAA Subscale (SA)	Metacognition MAS, IPII, BLERT	SA was associated with decreased metacognitive capacity	5
Mansueto et al. (2019) Netherlands	Psychotic disorder = 757 28, 25%	CTQ Composite & Subtypes (PA, EA, SA, PN, EN)	Mentalization, Social Cognition HT	PN and EN were associated with decreased mentalizing abilities, while PA, EA and SA were not; mentalization abilities partially mediated the association between PN and EN and negative and disorganized symptoms, especially in men; moderation analysis were not significant	7
Nazarov et al. (2014) Canada	PTSD = 31 42, 100% HC = 20 36, 100%	CTQ Composite	ToM IPT–15, RMET	CT is associated with lower ToM performance in women with PTSD	6
Ostefjells et al. (2017) Norway	BD and Psychotic disorder = 261 30, 46%	CTQ Composite & Subtypes (EA)	Metacognition MCQ–30-UD	The effect of EA on depressive/anxiety symptoms (but not on positive symptoms) was partially mediated by metacognitive impairments	7
Palmier-Claus et al. (2016) United Kingdom	SCZ = 20 40, 35% FEP = 20 25, 20% UHR = 14 23, 57% HC = 120 20, 71%	CTQ Composite	ToM HT, RMET	CT was not associated with ToM abilities	6
Parlar et al. (2014) Canada	PTSD = 29 43, 100% HC = 20 36%, 100%	CTQ Composite	Cognitive empathy, Mentalization IRI, TEQ	CT was not associated with cognitive empathy scores	5
Petersen et al. (2016) Australia	BPD = 19 33, 95% HC = 20 33, 95%	CAT Composite & Subtypes (Punishment, Neglect, SA)	Mentalization, ToM IRI, RMET, EAT, False-Belief Picture Sequencing Task, Joke-Appreciation task, Faux Pas test	Punishment was associated with decreased mentalization abilities while Neglect and SA were not	6
Quide et al. (2018) Australia	SCZ and Schizoaffective disorder = 79 43, 43% BD = 84 38, 64% HC = 75 36, 45%	CTQ Composite	Social Cognition, ToM FEEST, TASIT	CT is associated with deficits in social cognition	7
Rnic et al. (2018) Canada	MDD = 55 22, 69% HC = 70 22, 79%	CECA Composite & Subtypes (PA, EA, PN, EN)	ToM RMET	EA was associated with decreased ToM while PN and EN were associated with an increased ToM; PA was not associated with ToM impairments in the clinical population	7
Rokita et al. (2021) Ireland	SCZ and Schizoaffective disorder = 74 45, 32% HC = 116 35, 45%	CTQ Composite & Subtypes (PA, EA, SA, PN, EN)	Social Cognition, ToM HT, RMET	ToM performance was not associated with any CT subtype	6
Schalinski et al. (2018) USA	Psychotic disorder = 168 28, 33% HC = 50 27, 44%	MACE Composite & Subtypes (PA, EA, SA, PN, EN)	Social Cognition MSCEIT-ME	Social cognition deficits were associated with PN and EN occurring at age 11–12 and to cumulative CT	7
Simon et al. (2019) Hungary	MDD = 60 33, 75% HC = 32 33, 66%	CTQ Composite & Subtypes (PA, EA, SA, PN, EN) ETI Composite	ToM RMET	ToM performance was negatively impacted by EA, PN and EN but not by PA and SA; a negative cumulative effect of CT on ToM was also evidenced	6
Trauelsen et al. (2019) Denmark	FEP = 92 22, 27%	CTQ Composite & Subtypes (PA, EA, SA, PN, EN) CECA Subtypes (Separation, Institutionalization)	Metacognition MAS-A	EA, SA, and EN were associated with better metacognitive abilities, while no association was found for PA and PN	7
Vaskinn et al. (2021) Norway	SCZ and Schizoaffective disorder = 68 29, 37% HC = 70 29, 40%	CTQ Composite & Subtypes (PA, EA, SA, PN, EN)	ToM MASC	SA and PN were associated with decreased ToM performance, while PA, EA and EN were not	5
Weijers et al. (2018) Netherlands	Psychotic disorder = 87 32, 36%	CECA Composite	Mentalization HT	CT was associated with mentalization impairments, which mediated 40% of the association between CT and negative symptoms	6

*Abbreviations:* ACE, Adverse Childhood Experience questionnaire; BLERT, Bell-Lysaker Emotional Recognition Task; BD, Bipolar Disorders; BPD, Borderline Personality Disorder; CAT, Child Abuse and Trauma scale; CECA, Childhood Experience of Care and Abuse; CEAS, Childhood Emotional Abuse Scale; CHR, Clinical High Risk; CT, Childhood Trauma; CTQ, Childhood Trauma Questionnaire; EA, Emotional Abuse; EAT, Expression Attribution Test; EN, Emotional Neglect; ETI, Early Trauma Inventory; FEEST, Facial Expressions of Emotion: Stimuli and Tests; FEP, First Episode Psychosis; GP, General Population; HC, Healthy Controls; HT, Hinting Task; IPII, Indiana Psychiatric Illness Interview; IPT-15, Interpersonal Perception Task-15; IRI, Interpersonal Reactivity Index; MACE, Maltreatment and Abuse Chronology of Exposure scale; MAS, Metacognition Assessment Scale; MAS-A, Metacognition Assessment Scale-Abbreviated; MCQ-30, Metacognitions Questionnaire-30 items-Uncontrollability and Danger of thoughts; MDD, Major Depressive Disorder; MET, Multifaceted Empathy Task; MASC, The Movie for the Assessment of Social Cognition; MZQ, Mentalization Questionnaire; PA, Physical Abuse; PN, Physical Neglect; PTSD, Post Traumatic Stress Disorder; RFQ, Reflective Functioning Questionnaire; RFS, Reflective Functioning Scale; RMET, Reading the Mind in the Eyes Test; SA, Sexual Abuse; SCZ, Schizophrenia; SUD, Substance Use Disorder; TAA, Trauma Assessment for Adults; TASIT, The Awareness of Social Inference Test; TEQ, Toronto Empathy Questionnaire; ToM, Theory of Mind.

**Figure 2.**
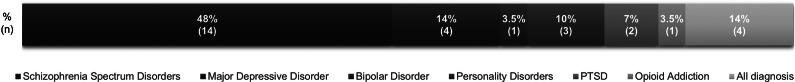
Clinical populations represented in the selected studies.

The studies were all published between 2011 and 2022, with 22 out of 29 being published between 2016 and 2020. The different clinical populations that the studies focused on can be seen in Figure 2.

All studies employed a cross-sectional design and recruited clinical populations from inpatient and outpatient mental health services. All diagnoses were based on the International Classification of Diseases (ICD-10) or the Diagnostic and Statistical Manual of Mental Disorders (DSM-IV and V) diagnostic criteria. The sample sizes of the clinical populations ranged from 19 to 757 patients. Of the 29 studies, 19 included healthy control groups, while only two studies reported well-matched controls (Chiesa & Fonagy, 2014; Brune, Walden, Edel, & Dimaggio, 2016).

The majority of the studies (*n* = 22, 76%) assessed CT with well-established measures, namely the Childhood Trauma Questionnaire (CTQ) (Bernstein et al., 2003) and the Childhood Experience of Care and Abuse (CECA) Scale (Bifulco, Bernazzani, Moran, & Jacobs, 2005). A total of nine studies (31%) employed a composite measure of CT, while most studies (*n* = 20, 69%) discriminated between the different subtypes of CT, namely physical abuse, emotional abuse, sexual abuse, physical neglect, and emotional neglect.

Details on mentalization domains across the studies are found in Supplementary Table S3. Mentalization abilities were assessed using a diverse array of tests, as also listed in Table 1. The Reading the Mind in the Eyes Test (RMET) (Baron-Cohen et al., 2001) and the Hinting Task (HT) (Corcoran, Mercer, & Frith, 1995) were the most frequently employed tests, each being used in six studies, while the Metacognition Assessment Scale-Abbreviated (MAS-A) (Lysaker et al., 2005) and the Mayer–Salovey–Caruso Emotional Intelligence Test-Managing Emotions (MSCEIT-ME) (Brackett & Salovey, 2006) were used in four studies each.

Only six studies conducted a mediation analysis with mentalization as a mediator between CT and clinical outcome (Chiesa & Fonagy, 2014; Belvederi Murri et al., 2017; Ostefjells et al., 2017; Weijers et al., 2018; Mansueto et al., 2019; Li et al., 2020). They focused on various clinical populations, and they all found that mentalization impairments partially mediated the effect of CT on the psychiatric disorders, especially on negative and depressive symptoms. Additionally, the study by Mansueto et al. (2019) is the only one that performed a moderation analysis, finding no moderating effect of mentalization on the impact of CT on clinical conditions in individuals with a psychotic disorder.

### Study quality assessment

The methodological quality of the included studies was assessed through the NOS scale and is presented in detail in Supplementary Table S4. The 29 included studies had scores ranging from 5 to 7 (poor to good), with 9 (31%) being rated as good quality, 14 (48%) as fair, and 6 as poor (21%). Most studies scored well on the ‘Selection’ domain, particularly in regard to the representativeness of the exposed cohorts and the ascertainment of trauma exposure. Nonetheless, many studies had limited comparability, with only few controlling for key confounders or including samples over 100 participants. Moreover, most of the included studies did not report *a priori* power analyses or otherwise justify their sample sizes. Finally, when considering the ‘outcome’ domains, if all the studies used validated measures, due to their cross-sectional design, they all had insufficient follow-up periods, which limited the ability to infer causality or long-term outcomes.

### Transdiagnostic association between childhood trauma and mentalization capacity

Over the 29 studies included in this systematic review, 20 (69%) found at least one analysis, examining any form of CT (composite or subtype), supporting the presence of a negative association between CT and mentalization. One single study found evidence of a positive association (Trauelsen et al., 2019), while eight (28%) studies reported no association between CT and mentalization. Notably, among the eight studies with no association, five utilized a composite measure of CT (Parlar et al., 2014; Andreou et al., 2015; Palmier-Claus et al., 2016; Addington et al., 2019; Guhn et al., 2020), one focused solely on abuse (Branas et al., 2022), and two assessed the five subtypes of CT (Jimenez et al., 2017; Rokita et al., 2021).

**Figure 3.**
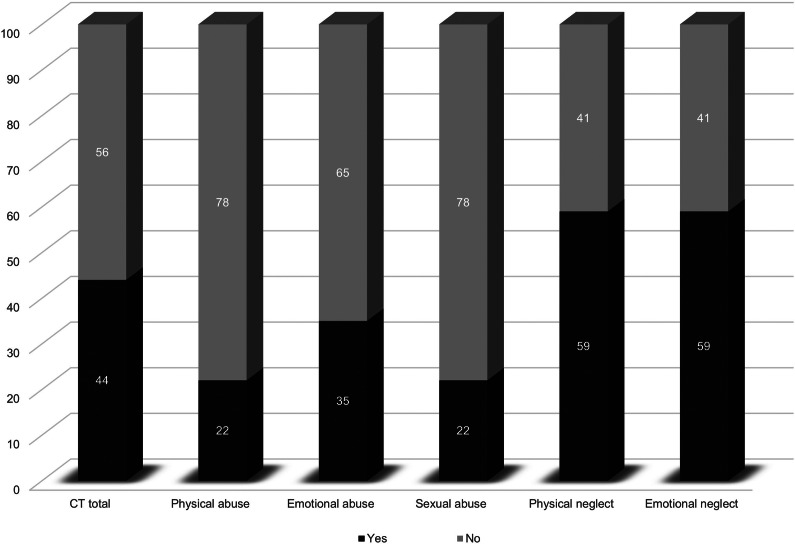
Association between CT and mentalization by subtypes of CT.

Figure 3 illustrates the percentage of studies that reported negative associations between various forms of CT and levels of mentalization. The subtypes of neglect – specifically physical and emotional neglect – were most commonly linked to mentalization deficits, with 59% of studies indicating a negative association. In contrast, the evidence for a negative relationship between abuse subtypes and mentalization was more limited; only 22% of studies on physical and sexual abuse and 35% of studies on emotional abuse found a negative association. When examining CT as a composite measure, 44% of studies reported a negative association with mentalization capacity.

### Diagnostic-specific associations between childhood trauma and mentalization capacity

#### Schizophrenia spectrum disorders

A significant portion of the studies (*n* = 14, 48%) focused on the relationship between CT and mentalization in the broad psychosis spectrum disorders, spanning from Clinical High-Risk for Psychosis to Schizophrenia Spectrum Disorders. Among these, nine studies identified a negative correlation, revealing that higher levels of CT were associated with reduced mentalization capacity. Specifically, mentalization deficits were associated with neglect (physical and emotional) in six studies (Garcia et al., 2016; Kilian et al., 2018; Kincaid et al., 2018; Schalinski, Teicher, Carolus, & Rockstroh, 2018; Mansueto et al., 2019; Vaskinn et al., 2021), sexual abuse in two studies (Lysaker Paul et al., 2011; Vaskinn et al., 2021), and emotional abuse in one study (Aydin et al., 2016); notably, no study established a specific link with physical abuse. In contrast, four studies reported no significant association between CT and mentalization abilities (Palmier-Claus et al., 2016; Addington et al., 2019; Rokita et al., 2021; Branas et al., 2022). It is interesting to notice that two utilized a composite measure of CT, and one focused solely on abuse without assessing neglect. Notably, Trauelsen et al. (2019) reported a positive correlation between different subtypes of CT and metacognition abilities.

#### Major depressive disorder

Four studies explored the link between CT and mentalization in patients with major depressive disorder (MDD). Among these, three studies reported a negative correlation (Rnic et al., 2018; Simon et al., 2019; Li et al., 2020), while one study found no significant association (Guhn et al., 2020).

#### Bipolar disorder

Research on bipolar disorder is limited, with only Jimenez et al. (Jimenez et al., 2017) investigating the connection between childhood adversities and mentalization capacity. This study did not find a significant association.

#### Personality disorders

Three studies (Chiesa & Fonagy, 2014; Brune et al., 2016; Petersen, Brakoulias, & Langdon, 2016) examined mentalization abilities in individuals diagnosed with personality disorders, all of which found a significant association with experiences of CT, indicating that early trauma adversely affects mentalization capacity in this group.

#### Post-traumatic stress disorder

Two studies addressed the relationship between mentalization abilities and CT in individuals with post-traumatic stress disorder (PTSD). The findings were conflicting: Nazarov et al. (2014) found a negative correlation, whereas Parlar et al. (2014) reported no significant association.

#### Opioid addiction

Eidenmueller et al. (2021) investigated ToM abilities in individuals diagnosed with opioid addiction who were undergoing substitution treatment. Their findings indicated a significant negative correlation between CT and mentalization capacity.

#### Heterogeneous disorders

Four studies examined the CT-mentalization association within a heterogeneous sample of psychiatric diagnoses. Andreou et al. (2015) compared individuals with borderline personality disorder and schizophrenia to a healthy control group, finding no evidence of an association between CT and mentalization. In contrast, Ostefjells et al. (2017) compared individuals with psychotic disorders to those with bipolar disorder, while Belvederi Murri et al. (2017) analyzed a general psychiatric population against healthy controls. Quide et al. (2018) also compared individuals with schizophrenia and bipolar disorder to healthy controls. All three studies identified a significant negative correlation between CT and mentalization in psychiatric populations.

### Association between childhood trauma and overlapping concepts used to refer to mentalization

#### Mentalization

Seven studies (24%) (Chiesa & Fonagy, 2014; Brune et al., 2016; Petersen et al., 2016; Belvederi Murri et al., 2017; Weijers et al., 2018; Mansueto et al., 2019; Li et al., 2020) focused specifically on mentalization, using a wide array of instruments for assessment (see Table 1). They all found a significant association between CT and mentalization impairments.

#### Theory of mind

Similarly, seven studies (24%) (Nazarov et al., 2014; Palmier-Claus et al., 2016; Kincaid et al., 2018; Rnic et al., 2018; Simon et al., 2019; Eidenmueller et al., 2021; Vaskinn et al., 2021) examined ToM, typically assessed via tasks such as the RMET and the HT. Six of these studies demonstrated a significant association between CT and ToM performances, while Palmier-Claus et al. found no link.

#### Metacognition

Metacognition was the focus of five studies (17%) (Lysaker Paul et al., 2011; Aydin et al., 2016; Ostefjells et al., 2017; Addington et al., 2019; Trauelsen et al., 2019), which mainly used the MAS-A to investigate it. Four studies supported a negative correlation between CT and metacognitive capacity. In contrast, Trauelsen et al. reported a paradoxical finding, with CT being associated with better metacognitive functioning in individuals with first-episode psychosis.

#### Social cognition

Eight studies (28%) (Andreou et al., 2015; Garcia et al., 2016; Jimenez et al., 2017; Kilian et al., 2018; Quide et al., 2018; Schalinski et al., 2018; Rokita et al., 2021; Branas et al., 2022) explored the broader concept of social cognition, yielding conflicting results regarding its association with CT: four studies identified a link, while four did not. These studies employed five different assessment tools, detailed in Table 1, with the MSCEIT-ME being the most frequently used.

#### Cognitive empathy

Finally, two studies (7%) (Parlar et al., 2014; Guhn et al., 2020) examined cognitive empathy, employing three different scales (see Table 1), and both reported no association with CT.

## Discussion

This systematic review aimed to synthesize the existing literature on the association between childhood trauma and mentalization within psychiatric populations. Our analysis identified 29 relevant studies, reflecting a growing but recent interest in this field, particularly over the last decade. Overall, our results support a significant relationship between childhood trauma exposure, particularly neglect, and mentalization deficits transdiagnostically.

Half of these studies focused on individuals with schizophrenia spectrum disorders, while the remaining half examined a variety of conditions, including MDD, bipolar disorder, PTSD, personality disorders, and opioid addiction. Although autism spectrum disorder, attention-deficit/hyperactivity disorder, and eating disorders were included in our search strategy, no eligible studies involving these populations were identified, suggesting a potential gap in the literature at the intersection of trauma, mentalization, and these specific diagnoses. Considering the uneven representation of clinical populations across the included studies, the observed strength of associations between childhood trauma, particularly neglect, and mentalization impairments may partially reflect the availability of research rather than true diagnostic differences. This imbalance limits the generalizability of our findings across all psychiatric diagnoses and highlights the need for more research in underrepresented populations.

Nevertheless, the findings mainly indicate a significant negative correlation between childhood trauma and impairments in mentalization abilities across clinical samples, with the strongest associations observed in those with schizophrenia, MDD, and personality disorders, and broadly a greater impact of neglect experiences as against to abuse. In accordance with the literature (Fonagy & Luyten, 2009; Ensink et al., 2017; Rodriguez et al., 2021; Martin-Gagnon et al., 2023), this suggests that experiences of childhood trauma may disrupt the normal development of mentalization, a critical social cognitive skill essential for understanding one’s own and others’ mental states.

Our finding of a stronger evidence linking neglect, as compared to abuse, with mentalizing abilities aligns with attachment theory, which posits that neglect can hinder the development of secure attachment necessary for self-reflection and interpersonal understanding (Bowlby, 1969; Fonagy & Target, 1997). The diminished frequency of meaningful interactions between caregivers and children can limit opportunities for the child to learn about emotional states and develop a coherent sense of self and others (Luyten et al., 2020). Such deficits in early relational experiences may have lasting impacts on social cognitive skills, potentially perpetuating mental health challenges in adulthood. Moreover, neurological evidence suggests distinct effects of different types of trauma on brain development (McLaughlin, Sheridan, & Lambert, 2014). For instance, neglect is associated with reduced amygdala volume, while abuse can lead to increased amygdala volume (Teicher, Samson, Anderson, & Ohashi, 2016). These structural changes may influence social cognition differently, underscoring the importance of differentiating among the subtypes of childhood adversity when studying their effects on social cognition.

While our review focused on mentalization-related constructs, it is also important to consider the broader cognitive impairments associated with childhood trauma. Recent meta-analyses have demonstrated robust associations between early trauma and deficits in attention, working memory, and executive functioning (Rodriguez et al., 2020; Vargas et al., 2019). These core cognitive domains may play a foundational role in supporting higher-order processes such as perspective-taking, a key component of mentalization. Consequently, impairments in basic cognitive functions could also moderate or mediate the impact of trauma on social cognitive development, and future studies should consider incorporating measures of general cognition to better elucidate their interplay.

The observed relationship between childhood trauma and mentalization impairments across disorders may also be understood in the context of the ‘P factor’ hypothesis (Caspi & Moffitt, 2018), which posits that various mental health conditions are interrelated and often rooted in common etiological pathways – such as adverse childhood experiences – thus aiming to explain the co-occurrence and heterogeneity of psychiatric symptoms. As mentalization plays a central role in self-regulation, interpersonal functioning, and adaptation to social stressors, and its impairments have been observed in multiple psychiatric conditions, sometimes even prior to illness onset, it might represent a transdiagnostic marker of this underlying vulnerability (Nolte et al., 2013; Luyten et al., 2020). However, the current evidence is still limited, and further longitudinal studies are needed to clarify the role of mentalization within this framework. Future research should test whether mentalization deficits predict a broad spectrum of psychiatric outcomes over time, and whether enhancing mentalization in early intervention contexts could mitigate the long-term impact of early trauma.

While the cross-sectional design of the reviewed studies poses limitations, preventing us from establishing causal relationships, the presence of a mediation effect in all the six studies that investigated this aspect, supports the hypothesis that mentalization may serve as a protective factor for those who have experienced childhood trauma and should be further studied in longitudinal prospective studies. This highlights the importance of considering mentalization in both research and clinical practice, particularly when developing interventions for individuals with a history of trauma. Concerning the assessment of childhood trauma, the majority of the studies used retrospective self-report tools (CTQ, CECA), which might be prone to recall bias. Nevertheless, more and more studies suggest that these reports are reliable (Bernstein et al., 2003; Read, van Os, Morrison, & Ross, 2005; Fisher et al., 2009; Simpson et al., 2019). Additionally, except for two studies (Kincaid et al., 2018; Schalinski et al., 2018), there was no information about the timing or the duration of the childhood adversity exposure, which might limit our understanding of these complex relationships (Fares-Otero & Schalinski, 2024). The variability in methods used to assess mentalization across studies complicates the interpretation of results as well. The same instruments were applied to assess different concepts, and the same concepts were evaluated with varying tests, contributing to the often-conflicting results observed across the literature and preventing from performing a meta-analysis. Furthermore, in most of the studies, different terms such as ‘mentalization’, ‘ToM’, ‘metacognition’, ‘cognitive empathy’, ‘reflective functioning’, and ‘social cognition’ were used interchangeably and as synonyms, contributing to the complexity of the interpretation of their findings. This heterogeneity, partly due to the lack of agreement on the conceptualization of these terms, limited the feasibility and interpretability of meta-analytic aggregation, preventing us from carrying out quantitative analysis. Variability across studies also arose from the wide range of adversities examined, some of which – such as abuse and neglect – are not directly comparable. Future meta-analytical research confirming our findings is needed. Moreover, longitudinal prospective studies are needed to clarify the nature of the relationships between childhood trauma and mentalization. Such studies should also account for neurocognitive functions, medication use, and other confounding factors that may impact mentalization performance, such as socioeconomic status, psychosocial stressors, supportive environment, and resilience. A transdiagnostic approach, as suggested by our findings, could enhance our understanding of mentalization as a universal target for intervention across various psychiatric disorders.

Given the role of mentalization as a common factor underlying effective psychosocial interventions (Fonagy & Allison, 2012), strengthening this capacity in therapeutic practices may be crucial for improving outcomes in patients with a history of trauma. One of the most studied approaches is MBT (Bateman, Fonagy, & Allen, 2009), originally developed for borderline personality disorder. MBT aims to enhance individuals’ ability to understand both their own and others’ mental states, particularly under emotional stress, by fostering reflection on interpersonal experiences and attachment-related responses – often disrupted in those who suffered from early trauma. Its application has since broadened to other clinical populations, namely to antisocial personality disorder (Bateman, 2022; Fonagy et al., 2025), eating disorders (Robinson et al., 2016), substance use disorders (Fuggle et al., 2021), and psychotic disorders (Weijers et al., 2021), as well as to family (Asen & Fonagy, 2012) and group therapy settings (Fonagy, Campbell, & Bateman, 2017). Another relevant intervention is the Metacognitive Reflection and Insight Therapy (MERIT) (Lysaker et al., 2011; Van Donkersgoed et al., 2014) developed specifically for individuals with schizophrenia (de Jong et al., 2019; Hasson-Ohayon et al., 2024). Unlike MBT, MERIT does not draw from a developmental or attachment-based model but shares the goal of enhancing self-reflection, psychological flexibility, and the ability to form complex and coherent representations of self and others. While originally designed for psychotic disorders, its principles may also benefit other clinical populations with social-cognitive impairments.

The growing implementation of interventions designed to foster mentalization skills – both in individual and group formats – reinforces the idea that enhancing this social cognitive ability may mitigate some of the adverse effects of childhood trauma on mental health.

## Conclusion

In conclusion, this review underscores the significant relationship between childhood trauma, particularly neglect, and mentalization deficits transdiagnostically. As research in this area advances, a deeper understanding of these dynamics will be essential for developing targeted interventions that not only address mental health symptoms but also promote healthier interpersonal functioning and resilience in those affected by childhood trauma, ultimately aiming to prevent the emergence of psychiatric disorders.

## Supporting information

Gorgellino et al. supplementary material Gorgellino et al. supplementary material

## Supplementary material

The supplementary material for this article can be found at http://doi.org/10.1017/S0033291725100822.
