# Supplementary material for: The shadow of trauma: impaired mentalization in clinical populations – a systematic review
Source: Psychol Med. 2025 Jul 8;55:e186. doi: 10.1017/S0033291725100822 (PMC12270273; doi:10.1017/S0033291725100822)
Supplement: Gorgellino et al. supplementary material [file S0033291725100822sup001.docx]

**Supplementary Material**

**The shadow of trauma: impaired mentalization in clinical populations—A systematic review.**

Marianna Gorgellino, Geetanjali Kumar, Yusra Parkar, Ana Catalan, Natalia E. Fares-Otero, Martin Debbané, Marco Armando, Luis Alameda.

**Table S1.** PRISMA Statement and Checklist

**Search Strategy**

- ***Childhood Trauma terms***
- ***Population/Diagnosis terms***
- ***Mentalization domain terms***

**Table S2.** Diagnostic manual codes

**Predictors** (details on childhood trauma assessments and operationalization)

**Outcomes** (details on mentalization outcomes assessments and operationalization)

**Table S3.** Operationalization of mentalization outcome measures of mentalization

**Quality Assessment procedures**

- **Table S4.** Newcastle-Ottawa Scale Quality assessment (NOS)
- ***Newcastle-Ottawa Scale***

**References**

**Table S1.** PRISMA Statement and Checklist.

| **Section/Topic** | **#** | **Checklist Item** | **Page** |
| --- | --- | --- | --- |
| **TITLE** | | | |
| Title | 1 | Identify the report as a systematic review, meta-analysis or both. | 1 |
| **ABSTRACT** | | | |
| Structured Summary | 2 | Provide a structured summary including as applicable: background; objectives; data sources; study eligibility criteria, participants, and interventions; study appraisal and synthesis methods; results; limitations; conclusions and implications of key findings; systematic review and registration number. | 2 |
| **INTRODUCTION** | | | |
| Rationale | 3 | Describe the rationale for the review in the context of existing knowledge. | 2-3 |
| Objectives | 4 | Provide an explicit statement of objective(s) or question(s) the review addresses with reference to participants, interventions, comparison, outcomes, and study design (PICOS). | 2-3 |
| **METHODS** | | | |
| Eligibility Criteria | 5 | Specify the inclusion and exclusion criteria for the review and how studies were grouped for syntheses. | 4 |
| Information Sources | 6 | Specify all databases, registers, websites, organisations, reference lists and other sources searched or consulted to identify studies. Specify the date when each source was last searched or consulted. | 4 |
| Search Strategy | 7 | Present full electronic search strategy for at least one database, including any limits used, such that it could be repeated. | SM |
| Selection Process | 8 | State the process for selecting studies (i.e. screening, eligibility, included in systematic review, and if applicable, included in meta-analysis). | 4 |
| Data Collection Process | 9 | Specify the methods used to collect data from reports, including how many reviewers collected data from each report, whether they worked independently, any processes for obtaining or confirming data from study investigators, and if applicable, details of automation tools used in the process. | 4 |
| Data Items | 10a | List and define all outcomes for which data were sought. Specify whether all results that were compatible with each outcome domain in each study were sought (e.g. for all measures, time points, analyses), and if not, the methods used to decide which results to collect. | 4 and SM |
|  | 10b | List and define all other variables for which data were sought (e.g. participant and intervention characteristics, funding sources). Describe any assumptions made about any missing or unclear information. | 4 and SM |
| Study Risk of Bias Assessment | 11 | Specify the methods used to assess risk of bias in the included studies, including details of the tools used, how many reviewers assessed each study and whether they worked independently, and if applicable, details of automation tools used in the process. | 4 and SM |
| Effect Measures | 12 | Specify for each outcome the effect measures (e.g. risk ratio, mean difference) used in the synthesis or presentation of results. | 4 |
| Synthesis Methods | 13a | Describe the processes used to decide which studies were eligible for each synthesis, (e.g. tabulating the study intervention characteristics and comparing against the planned groups for synthesis). | 4 and SM |
|  | 13b | Describe any methods required to prepare the data for presentation or synthesis, such as handling of missing summary statistics, or data conversion. | 4 |
|  | 13c | Describe any methods used to tabulate or visually display results of individual studies and synthesis. | 4 and SM |
|  | 13d | Describe any methods used to synthesise results and provide rationale for the choices. If meta-analysis was performed, describe the models, methods to identify the presence and extent of statistical heterogeneity, and software packages used. | 4 and SM |
|  | 13e | Describe any methods used to explore possible causes of heterogeneity among study results (e.g. subgroup analyses, meta-regression). | 4 |
|  | 13f | Describe any sensitivity analyses conducted to assess robustness of the synthesised results. | 4 |
| Reporting Bias Assessment | 14 | Describe any methods used to assess risk of bias due to missing results in a synthesis (arising from reporting biases). | 4 |
| Certainty Assessment | 15 | Describe any methods used to assess certainty (or confidence) in the body of evidence for an outcome. | 4 and SM |
| **RESULTS** | | | |
| Study Selection | 16a | Describe the results of the search and selection process, from the number of records identified in the search to the number of studies included in the review, ideally using a flow diagram. | 5 and fig.1 |
|  | 16b | Cite studies that might appear to meet inclusion criteria, but which were excluded, and explain why they were excluded. | 5 and fig.1 |
| Study Characteristics | 17 | Cite each included study and present its characteristics. | 5-7 and Table 1 |
| Risk of Bias In Studies | 18 | Present assessment of risk of bias for each included study. | 5 and SM |
| Results in Individual Studies | 19 | For all outcomes, present, for each study: (a) summary statistics for each group (where appropriate) and (b) an effect estimate and its precision (e.g. confidence/credible intervals), ideally using structured tables and plots. | 5-7 and Table 1 |
| Results of Synthesis | 20a | For each synthesis, briefly summarise the characteristics and risk of bias among contributed studies. | 5-7 and SM |
|  | 20b | Present results of all statistical syntheses conducted. If meta-analyses was done, present for each the summary estimate and its precision (e.g. confidence/credible interval) and measures of statistical heterogeneity. If comparing groups, describe the direction of the effect. | 5-7 and Table 1 |
|  | 20c | Present results of all investigations of possible causes of heterogeneity among study results. | 6-7 and SM |
|  | 20d | Present results of all sensitivity analyses conducted to assess the robustness of the synthesised results. | 6-7 and SM |
| Reporting Bias | 21 | Present assessments of risk of bias due to missing results (arising from reporting biases) for each synthesis assessed. | Table 1 and SM |
| Certainty of Evidence | 22 | Present assessments of certainty (or confidence) in the body of evidence for each outcome assessed. | Table 1 and SM |
| **DISCUSSION** | | | |
| Discussion | 23a | Provide a general interpretation of the results in the context of other evidence. | 7-9 |
|  | 23b | Discuss any limitations of the evidence included in the review. | 8 |
|  | 23c | Discuss any limitation of the review processes used. | 8 |
|  | 23d | Discuss implications of the results for practice, policy, and future research. | 8-9 |
| **OTHER INFORMATION** | | | |
| Registration and protocol | 24a | Describe sources of funding for the systematic review and other support; role of funders for the systematic review. | 9 |
|  | 24b | Indicate where the review protocol can be accessed, or state that a protocol was not prepared. | 3 |
|  | 24c | Describe and explain any amendments to information provided at registration or in the protocol. | NA |
| Support | 25 | Describe sources of financial or non-financial support for the review, and the role of the funders or sponsors in the review. | 9 |
| Competing Interests | 26 | Declare any competing interests of review authors. | 9 |
| Availability of Data, Code, and Other Materials | 27 | Report which of the following are publicly available and where they can be found: template data collection forms; data extracted from included studies; data used for all analyses; analytic code; any other materials used in the review. | 9 |

*Note.* Adapted from the PRISMA 2020 Statement (Page et al., 2021).

**Search Strategy**

(Embase, Medline and PsycInfo databases)

**Childhood Trauma terms:**

Exp Sex Offenses

Exp Physical Abuse

Exp Child Abuse

Exp Bullying

Exp Sexual Abuse

Exp Physical Abuse

Exp Emotional Abuse

Exp Child Abuse

Exp Child Neglect

Exp Emotional Trauma

Exp Bullying

Exp Abandonment

Exp Rape

Exp Domestic Violence

Exp Sexual Harassment

Exp Rape

Exp Domestic Violence

Exp Sexual Harassment

Exp sexual abuse

Exp physical abuse

Exp emotional abuse

Exp neglect

Exp child abuse

Exp bullying

Exp sexual bullying

Exp rape

Exp domestic violence

Exp psychotrauma

Exp sexual harassment

Exp emotional abuse

Exp parental deprivation

Exp rape

Exp childhood adversity

(separat* adj5 parent*).mp

victimi*.mp.

(advers* adj5 experienc*).mp

adversit*.mp

emotional abuse.mp

psychological abuse.mp

neglect.mp

bully*.mp

bullied*.mp

parental loss.mp

(Childhood adj5 trauma).mp

(abandon adj10 parent*).mp

maltreat*.mp

(Parent adj5 loss).mp

(Childhood adj5 maltreat*).mp

early adversity.mp

being taken into care.mp

rape.mp

domestic violence.mp

sexual harassment.mp

sexual abuse.mp

physical abuse.mp

"childhood adversity".mp

**Population/Diagnosis terms:**

Exp psychosis

Exp schizophrenia

Exp schizoaffective psychosis

Exp Psychotic Disorders

Exp Schizophrenia

Exp Psychosis

Exp Acute Psychosis

Exp Affective Psychosis

Exp Schizophrenia

Exp Schizoaffectif Disorder

psychot*.mp

schizophr*.mp

schizoaf*.mp

Exp schizotypal personality disorder

Exp Schizotypal Personality Disorder

Exp Schizotypy

schizotyp*.mp

"at risk mental state".mp

"psychosis risk".mp

(prodrom* adj3 psychosis).mp

"ultra high risk".mp

"clinical high risk".mp

Exp mental disease

Exp depression

Exp major depression

Exp anxiety

Exp anxiety disorder

Exp bipolar disorder

Exp posttraumatic stress disorder

Exp eating disorder

Exp autism

Exp attention deficit hyperactivity disorder

Exp impulsiveness

Exp alcoholism

Exp alcohol abuse

Exp substance abuse

Exp drug dependence

Exp drug abuse

Exp Mental Disorders

Exp Depression

Exp Depressive Disorder

Exp Anxiety

Exp Anxiety Disorders

Exp Bipolar Disorder

Exp Stress Disorders, Post-Traumatic

Exp "Feeding and Eating Disorders"

Exp Autistic Disorder

Exp Autism Spectrum Disorder

Exp Attention Deficit Disorder with Hyperactivity

Exp Impulsive Behaviour

Exp Alcoholism

Exp Substance-Related Disorders

Exp Mental Disorders

Exp Major Depression

Exp Anxiety

Exp Anxiety Disorders

Exp Bipolar Disorder

Exp Posttraumatic Stress Disorder

Exp Eating Disorders

Exp Autism Spectrum Disorders

Exp Attention Deficit Disorder with Hyperactivity

Exp Impulsiveness

Exp Alcoholism

Exp Alcohol Abuse

Exp Drug Abuse

depression.mp

depressive disorder.mp

major depressive disorder.mp

psychopathology.mp

mood disorders.mp

"major depressive disorder".mp

"mood disorder".mp

"mood depression".mp

MDD.mp

anxiety.mp

"anxiety disorder".mp

bipolar.mp

mani*.mp

cyclothymi*.mp

manic-depressi*.mp

hypomani*.mp

"bipolar affective disorder".mp

PTSD.mp

"post traumatic stress disorder".mp

"posttraumatic stress".mp

"eating disorder".mp

anorexia.mp

bulimia.mp

"binge eating".mp

autism.mp

"autistic disorder".mp

"autism spectrum disorder".mp

disorder, autistic.mp

"attention deficit hyperactivity disorder".mp

ADHD.mp

hyperkinetic.mp

"hyperkinetic disorder".mp

alcoholism.mp

"alcohol abuse".mp

"alcohol dependence".mp

"mental health".mp

(mental health adj3 disorder).mp

psychiatr*.mp

(psychiatry* adj3 disorder*).mp

(mental health adj3 disorder*).mp

(mental health adj3 condition).mp

"psychiatr* diagnos*".mp

**Mentalization domain terms:**

Exp mentalization

Exp "theory of mind"

Exp metacognition

Exp social cognition

ToM.mp

mentalis*.mp

mentalization.mp

metacogn*.mp

mentaliz*.mp

"theory of mind"

"social cognition".mp

mind reading.mp

mind mindedness.mp

Exp Mindfulness

reflective functioning.mp

Exp Reflectiveness

Mindfulness.mp

**Filters applied to the final search:**

- Duplication

- Not written in English

- Not human samples

**Table S2.** Diagnostic manual codes.

| **Diagnosis** | **Code used within ICD-10 (World Health, 2004)** | **Code used within DSM-V (American Psychiatric Association & American Psychiatric Association, 2013)** |
| --- | --- | --- |
| **Schizophrenia** | F20 | 295.90 |
| **Psychosis not otherwise specified** | F29 | 298.9 |
| **Major Depressive Disorder** | F32.9 | 296.20-296.36 |
| **Persistent Depressive Disorder** | F34.1 | 300.40 |
| **Bipolar Disorder** | F31.1 | 296.04/296.44/296.54/296.64 |
| **Anxiety Disorder** | F41.9 | 300.02 |
| **Post-traumatic stress disorder** | F43.1 | 309.81 |
| **Personality Disorders** | F60 | 301.90 |
| **Borderline Personality Disorder** | F60.3 | 301.83 |
| **Substance use disorders** | F55 | 305.00/304.00/304.20/304.30 |

*Note.* Only DSM-V codes are reported, some studies used DSM-IV diagnosis.

**Predictors** (details on childhood trauma assessments and operationalization)

CT as predictor was defined based on previous definitions (Arias et al., 2008; Gilbert et al., 2009), as at least one of the following: (a) CT composite - cumulative scores of different traumas whereby subtypes selected is dependent on study or a binary grouping of exposure to any trauma subtypes; (b) sexual abuse (SA), as sexual acts, including intercourse or touching etc. toward a child; (c) physical abuse (PA), as acts of violence causing physical harm or injury, including physical punishment; (d) emotional abuse (EA), as verbal or behavioural assaults toward a child that might result in trauma, including any demeaning or humiliating name-calling by an adult or caregiver; (e) physical neglect (PN), as caregivers’ failure to provide basic physical needs for the child, including shelter, food, clothing or health-care etc.; (f) emotional neglect (EN), as caregivers’ failure to meet the child’s fundamental emotional and psychological needs, including love, care, support, and belonging etc.

**Outcomes**(details on mentalization outcomes assessments and operationalization)

Mentalization is a complex construct that can be defined with various overlapping terms. In the current study, following an expert consensus (MA and MD) mentalization was considered as: (a) Mentalization (social cognitive imaginative activities that enable the interpretation of behaviours as intentional mental states and to imagine and understand their own mental states, as well as those of others (Fonagy et al., 2002)) (b) Theory of Mind (part of social cognition, the capacity to understand, predict, explain and infer others mental states (Goldman, 2012)), (c) Metacognition (ability to notice our own mental experiences, such as thoughts and emotions, and to synthetize them into a coherent sense of the self and others (Lysaker et al., 2013)), (d) Mindfulness (characterised by deliberate awareness which aims to divert attention to the present moment without judgment (Kabat-Zinn, 2003)), (e) Reflective Functioning (self and others relationship representation in the context of attachment theory (Fonagy et al., 1998)), (f) Social Cognition (sum of mental processes that underlie the perception and interpretation of stimuli pertinent to social interactions (Green et al., 2008); it includes emotion recognition and ToM (Mitchell & Phillips, 2015)), (e) Cognitive Empathy (the component of empathy that defines the ability to understand another person’s mental state (Schreiter et al., 2013)).

**Table S3.** Operationalization of mentalization.

| **Instruments** | **Domains used to operationalise** | **Utilised by** | **Total number of studies** |
| --- | --- | --- | --- |
| **HT**  **(Corcoran et al., 1995)** | 10 short vignettes depicting scenarios involving two people; each story is read, then the participant is asked about what the character really meant to say; if the answer is wrong, a hint is given | (Branas et al., 2022; Kincaid et al., 2018; Mansueto et al., 2019; Palmier-Claus et al., 2016; Rokita et al., 2021; Weijers et al., 2018) | 6 |
| **IRI**  **(Davis, 1983)** | **4 Domains:** Perspective taking, fantasy, empathetic concern, personal distress | (Guhn et al., 2020; Parlar et al., 2014; Petersen et al., 2016) | 3 |
| **MAS-A**  **(Semerari et al., 2003)** | **4 Domains:** Self Reflectivity (Understanding own mental states), Understanding of others Mind, Decentration (understanding that other people can have different motives and intentions), Mastery (using own thoughts to solve problems) | (Addington et al., 2019; Aydin et al., 2016; Lysaker Paul et al., 2011; Trauelsen et al., 2019) | 4 |
| **MASC**  **(Dziobek et al., 2006)** | a movie where four characters interact in social context is shown; participants are asked questions about the emotions, thoughts and intentions of the characters ; **6 Domains:** Total MASC score, Overmentalizing, Undermentalizing, Affective, Cognitive, no errors in mentalizing | (Andreou et al., 2015; Eidenmueller et al., 2021; Vaskinn et al., 2021) | 3 |
| **MSCEIT-ME**  **(Brackett & Salovey, 2006)** | participants read a short story about another person, and then determine how effective several different courses of action would be in coping with the emotions described in the story; participants rate a number of possible actions ranging from «very ineffective», to «very effective» | (Garcia et al., 2016; Jimenez et al., 2017; Kilian et al., 2018; Schalinski et al., 2018) | 4 |
| **RMET**  **(Baron-Cohen et al., 2001)** | 36 images of people's eyes region of the face are shown, and participants are asked to choose one word out of four to best describe what the person in the picture is thinking or feeling | (Nazarov et al., 2014; Palmier-Claus et al., 2016; Petersen et al., 2016; Rnic et al., 2018; Rokita et al., 2021; Simon et al., 2019) | 6 |

***Other Mentalization measures***

BLERT (Bell et al., 1997)

EAT (Langdon et al., 2006)

False-Belief Picture Sequencing Task (Langdon & Coltheart, 1999)

Faux Pas task (Baron-Cohen et al., 1997)

FEEST (Aw et al., 2002)

IPII (Lysaker et al., 2002)

IPT-15 (Costanzo & Archer, 1989)

Joke-Appreciation task (Langdon et al., 2010)

MAS (Semerari et al., 2003)

MCQ-30 (Wells & Cartwright-Hatton, 2004)

MET (Dziobek et al., 2008)

MZQ (Hausberg et al., 2012)

Novel Cartoon Task (Brune et al., 2016)

RFQ (Fonagy et al., 2016)

RFS (Fonagy et al., 1998)

TASIT (McDonald et al., 2003)

TEQ (Spreng et al., 2009)

**Quality Assessment procedures**

**Table S4.** Newcastle-Ottawa Scale Quality assessment (NOS) adapted.

| **Author** | **Selection** | | | | | **Comparability** | | **Outcome** | | | | **Total/**  **Quality**  **Classification** |
| --- | --- | --- | --- | --- | --- | --- | --- | --- | --- | --- | --- | --- |
|  | 1. Represen-  tativeness of exposed cohort | 2.  Selection of the non-exposed cohort | 3.  Ascertainment of exposure | 4.  Outcome of interest was not present at start of study | Domain  Score  (out of 4) | 1.  Comparability of cohorts  on the  basis of the design  or analysis | Domain Score  (out of 2) | 1.  Assessment of outcome | 2.  Adequate length of follow-up | 3.  Adequate follow-up of cohorts | Domain Score  (out of 3) |  |
| *Addington et al., (2019)* | * | * | * |  | 3/4 |  | 0/2 | * | * |  | 2/3 | 5/  Poor |
| *Andreou et al.,*  *(2015)* | * | * | * |  | 3/4 | * | 1/2 | * | * |  | 2/3 | 6/  Fair |
| *Aydin et al.,*  *(2016)* | * | * | * |  | 3/4 | * | 1/2 | * | * |  | 2/3 | 6/  Fair |
| *Belvederi Murri et al., (2017)* | * | * | * |  | 3/4 | * | 1/2 | * | * |  | 2/3 | 6/  Fair |
| *Branas et al.,*  *(2022)* | * | * | * |  | 3/4 | * | 1/2 | * | * |  | 2/3 | 6/  Fair |
| *Brüne et al.,*  *(2016)* | * | * | * |  | 3/4 | * | 1/2 | * | * |  | 2/3 | 6/  Fair |
| *Chiesa et al.,*  *(2014)* | * | * | * |  | 3/4 | ** | 2/2 | * | * |  | 2/3 | 7/  Good |
| *Eidenmueller et al.,*  *(2021)* | * | * | * |  | 3/4 |  | 0/2 | * | * |  | 2/3 | 5/  Poor |
| *Garcia et al.,*  *(2016)* | * | * | * |  | 3/4 | * | 1/2 | * | * |  | 2/3 | 6/  Fair |
| *Guhn et al.,*  *(2020)* | * | * | * |  | 3/4 | ** | 2/2 | * | * |  | 2/3 | 7/  Good |
| *Jiménez et al.,*  *(2017)* | * | * | * |  | 3/4 | ** | 2/2 | * | * |  | 2/3 | 7/  Good |
| *Killian et al.,*  *(2017)* | * | * | * |  | 3/4 |  | 0/2 | * | * |  | 2/3 | 5/  Poor |
| *Kincaid et al.,*  *(2018)* | * | * | * |  | 3/4 | * | 1/2 | * | * |  | 2/3 | 6/  Fair |
| *Li et al.,*  *(2020)* | * | * | * |  | 3/4 | * | 1/2 | * | * |  | 2/3 | 6/  Fair |
| *Lysaker et al.,*  *(2011)* | *** |  | * |  | 2/4 | * | 1/2 | * | * |  | 2/3 | 5/  Poor |
| *Mansueto et al., (2019)* | *** | * | * |  | 3/4 | ** | 2/2 | * | * |  | 2/3 | 7/  Good |
| *Nazarov et al.,*  *(2014)* | * | * | * |  | 3/4 | * | 1/2 | * | * |  | 2/3 | 6/  Fair |
| *Ostefjells et al.,*  *(2017)* | * | * | * |  | 3/4 | ** | 2/2 | * | * |  | 2/3 | 7/  Good |
| *Palmier - Claus et al.,*  *(2016)* | * | * | * |  | 3/4 | * | 1/2 | * | * |  | 2/3 | 6/  Fair |
| *Parlar et al.,*  *(2014)* | * | * | * |  | 3/4 |  | 0/2 | * | * |  | 2/3 | 5/  Poor |
| *Petersen et al.,*  *(2016)* | * | * | * |  | 3/4 | * | 1/2 | * | * |  | 2/3 | 6/  Fair |
| *Quidé et al.,*  *(2018)* | * | * | * |  | 3/4 | ** | 2/2 | * | * |  | 2/3 | 7/  Good |
| *Rnic et al.,*  *(2018)* | * | * | * |  | 3/4 | ** | 2/2 | * | * |  | 2/3 | 7/  Good |
| *Rokita et al.,*  *(2021)* | * | * | * |  | 3/4 | * | 1/2 | * | * |  | 2/3 | 6/  Fair |
| *Schalinski et al., (2018)* | * | * | * |  | 3/4 | ** | 2/2 | * | * |  | 2/3 | 7/  Good |
| *Simon et al.,*  *(2019)* | * | * | * |  | 3/4 | * | 1/2 | * | * |  | 2/3 | 6/  Fair |
| *Trauelsen et al.,*  *(2019)* | * | * | * |  | 3/4 | ** | 2/2 | * | * |  | 2/3 | 7/  Good |
| *Vaskinn et al.,*  *(2021)* | * | * | * |  | 3/4 |  | 0/2 | * | * |  | 2/3 | 5/  Poor |
| *Weijers et al.,*  *(2018)* | * | * | * |  | 3/4 | * | 1/2 | * | * |  | 2/3 | 6/  Fair |

***Newcastle Ottawa Scale***

Two independent reviewers (GK and YP) used the Newcastle-Ottawa Scale (NOS) for cohort studies to assess the quality of included studies; assessments were cross-checked by a third reviewer (LA). Studies were assessed based on 3 broad domains: 1) selection of participants (representativeness of exposed cohorts; selection of non-exposed cohort; ascertainment of exposure; and demonstration that outcome of interest was not present at start of study- this item was not applicable to this systematic review); 2) Comparability of cohorts (appropriate control for confounders; and a sample size of over 100 participants); 3) Outcome (assessment of outcome based on record linkage or formal interview of symptoms/scales; adequate follow-up for outcomes to occur; and adequate follow-up of cohorts with an attrition rate of below 20%). The maximum number of scores for each domain was 4 for Selection, 2 for Comparability, and 3 for Outcome. Total quality scores ranged from 0 to 9, with a higher score representing better quality. We rated the overall quality according to specific combinations of results across the 3 domains, following another published meta-analysis (Sharmin et al., 2017). Studies scoring 3 points in selection, 2 points in comparability, and 3 points in outcome were classified as ‘very good’ Studies scoring 3 points in selection, 2 points in comparability, and 1 or 2 points in outcome were classified as “good” quality. Studies scoring 3 points in Selection, 1 point in Comparability, and 1 or 2 points in Outcome were classified as “fair” quality. “Poor” quality studies scored 3 points in Selection, 0 points in Comparability, and 1 or 2 points in Outcome. Specific definitions of each domain and scoring of the NOS assessment can be found on Table S6 (SM).

Note: A study can be awarded a maximum of one star for each numbered item within the Selection and Outcome categories. A maximum of two stars can be given for Comparability.

**Selection**

1) Representativeness of the exposed cohort

a) truly representative of the average individuals with psychosis or attenuated psychotic symptoms in the community *

b) somewhat representative of the average individuals with psychosis or attenuated psychotic symptoms in the community *

c) selected group of users eg. nurses, volunteers

d) no description of the derivation of the cohort

2) Selection of the non-exposed cohort (are people with trauma drawn from the same population than the non-exposed?)

a) drawn from the same community/clinical population as the exposed cohort *

b) drawn from a different source

c) no description of the derivation of the non-exposed cohort

3) Ascertainment of exposure

a) secure record*

b) structured or semi-structured interview (such as CECA)*

c) written self-report (such as CTQ)* (star included here given the common use of self-reports in the field of adversity in psychosis)

d) no description

4) Demonstration that outcome of interest was not present at start of study (such as in birth cohorts such as ALSPAC or Twin Study, or 22Q11, or also in prospective cohort studies with two time points)

a) yes *

b) no

**Comparability**

1) Comparability of cohorts on the basis of the design or analysis

a) study controls for confounders (yes o no)*

b) study controls for any additional factor (sample above 100)*

**Outcome**

1) Assessment of outcome

a) independent blind assessment *

b) record linkage or formal interview of symptoms *

c) self-report

d) no description

2) Was follow-up long enough for outcomes to occur

a) yes *

b) no

3) Adequacy of follow up of cohorts (check in papers such as mine from CH, whether the follow-up lost (drop outs) is big (>20%)

a) complete follow up - all subjects accounted for *

b) subjects lost to follow up unlikely to introduce bias - small number lost < 20 % *

c) no statement/ no follow up

Note: we removed response c to be given a star also to studies with drop outs.

**References**

Addington, J., Shakeel, M. K., Braun, A., Bonneville, D., & Stowkowy, J. (2019). Metacognition in youth at-risk for psychosis. *Schizophrenia Research*, *210*, 303-305. https://doi.org/https://doi.org/10.1016/j.schres.2019.07.005

American Psychiatric Association, p., & American Psychiatric Association, D. S. M. T. F. a. (2013). *Diagnostic and statistical manual of mental disorders : DSM-5™* (5th edition. ed.). American Psychiatric Publishing, a division of American Psychiatric Association.

Andreou, C., Kelm, L., Bierbrodt, J., Braun, V., Lipp, M., Yassari, A. H., & Moritz, S. (2015). Factors contributing to social cognition impairment in borderline personality disorder and schizophrenia. *Psychiatry Res*, *229*(3), 872-879. https://doi.org/10.1016/j.psychres.2015.07.057

Arias, I., Leeb, R. T., Melanson, C., Paulozzi, L. J., & Simon, T. R. (2008). Child maltreatment surveillance; uniform definitions for public health and recommended data elements.

Aw, Y., Perrett, D., Calder, A., Sprengelmeyer, R., & Ekman, P. (2002). Facial expressions of emotion: Stimuli and tests (FEEST). *Thames Valley Test Company (TVTC)*.

Aydin, O., Balikci, K., Tas, C., Aydin, P. U., Danaci, A. E., Brune, M., & Lysaker, P. H. (2016). The developmental origins of metacognitive deficits in schizophrenia. *Psychiatry Research*, *245*, 15-21.

Baron-Cohen, S., Jolliffe, T., Mortimore, C., & Robertson, M. (1997). Another advanced test of theory of mind: evidence from very high functioning adults with autism or asperger syndrome. *J Child Psychol Psychiatry*, *38*(7), 813-822. https://doi.org/10.1111/j.1469-7610.1997.tb01599.x

Baron-Cohen, S., Wheelwright, S., Hill, J., Raste, Y., & Plumb, I. (2001). The "Reading the Mind in the Eyes" Test revised version: a study with normal adults, and adults with Asperger syndrome or high-functioning autism. *J Child Psychol Psychiatry*, *42*(2), 241-251.

Bell, M., Bryson, G., & Lysaker, P. (1997). Positive and negative affect recognition in schizophrenia: a comparison with substance abuse and normal control subjects. *Psychiatry Res*, *73*(1-2), 73-82. https://doi.org/10.1016/s0165-1781(97)00111-x

Brackett, M. A., & Salovey, P. (2006). Measuring emotional intelligence with the Mayer-Salovery-Caruso Emotional Intelligence Test (MSCEIT). *Psicothema*, *18*(Suppl), 34-41.

Branas, A., Lahera, G., Barrigon, M. L., Canal-Rivero, M., & Ruiz-Veguilla, M. (2022). Effects of childhood trauma on facial recognition of fear in psychosis [Efectos del trauma infantil en el reconocimiento de la expresion facial de miedo en psicosis.]. *Revista de Psiquiatria y Salud Mental*, *15(1)*, 29-37.

Brune, M., Walden, S., Edel, M. A., & Dimaggio, G. (2016). Mentalization of complex emotions in borderline personality disorder: The impact of parenting and exposure to trauma on the performance in a novel cartoon-based task. *Comprehensive Psychiatry*, *64*, 29-37.

Corcoran, R., Mercer, G., & Frith, C. D. (1995). Schizophrenia, symptomatology and social inference: Investigating “theory of mind” in people with schizophrenia. *Schizophrenia Research*, *17*(1), 5-13. https://doi.org/https://doi.org/10.1016/0920-9964(95)00024-G

Costanzo, M., & Archer, D. (1989). Interperting the expressive behavior of others: The Interpersonal Perception Task. *Journal of Nonverbal Behavior*, *13*(4), 225-245. https://doi.org/10.1007/BF00990295

Davis, M. H. (1983). Measuring individual differences in empathy: Evidence for a multidimensional approach. *Journal of Personality and Social Psychology*, *44*(1), 113-126. https://doi.org/10.1037/0022-3514.44.1.113

Dziobek, I., Fleck, S., Kalbe, E., Rogers, K., Hassenstab, J., Brand, M., Kessler, J., Woike, J. K., Wolf, O. T., & Convit, A. (2006). Introducing MASC: A Movie for the Assessment of Social Cognition. *Journal of Autism and Developmental Disorders*, *36*(5), 623-636. https://doi.org/10.1007/s10803-006-0107-0

Dziobek, I., Rogers, K., Fleck, S., Bahnemann, M., Heekeren, H. R., Wolf, O. T., & Convit, A. (2008). Dissociation of Cognitive and Emotional Empathy in Adults with Asperger Syndrome Using the Multifaceted Empathy Test (MET). *Journal of Autism and Developmental Disorders*, *38*(3), 464-473. https://doi.org/10.1007/s10803-007-0486-x

Eidenmueller, K., Grimm, F., Hermann, D., Frischknecht, U., Montag, C., Dziobek, I., Kiefer, F., & Bekier, N. K. (2021). Exploring Influences on Theory of Mind Impairment in Opioid Dependent Patients. *Frontiers in Psychiatry*, *12 (no pagination)*, Article 721690.

Fonagy, P., Gergely, G., Jurist, E. L., & Target, M. (2002). *Affect regulation, mentalization, and the development of the self*. Other Press.

Fonagy, P., Luyten, P., Moulton-Perkins, A., Lee, Y.-W., Warren, F., Howard, S., Ghinai, R., Fearon, P., & Lowyck, B. (2016). Development and Validation of a Self-Report Measure of Mentalizing: The Reflective Functioning Questionnaire. *PLoS ONE*, *11*(7), e0158678. https://doi.org/10.1371/journal.pone.0158678

Fonagy, P., Target, M., Steele, H., & Steele, M. (1998). REFLECTIVE-FUNCTIONING MANUAL Version 5 FOR APPLICATION TO ADULT ATTACHMENT INTERVIEWS.

Garcia, M., Montalvo, I., Creus, M., Cabezas, A., Sole, M., Algora, M. J., Moreno, I., Gutierrez-Zotes, A., & Labad, J. (2016). Sex differences in the effect of childhood trauma on the clinical expression of early psychosis. *Comprehensive Psychiatry*, *68*, 86-96.

Gilbert, R., Widom, C. S., Browne, K., Fergusson, D., Webb, E., & Janson, S. (2009). Burden and consequences of child maltreatment in high-income countries. *Lancet*, *373*(9657), 68-81. https://doi.org/10.1016/s0140-6736(08)61706-7

Goldman, A. (2012). Theory of Mind. In E. Margolis, R. Samuels, & S. P. Stich (Eds.), *The Oxford Handbook of Philosophy of Cognitive Science*. Oxford University Press.

Green, M. F., Penn, D. L., Bentall, R., Carpenter, W. T., Gaebel, W., Gur, R. C., Kring, A. M., Park, S., Silverstein, S. M., & Heinssen, R. (2008). Social cognition in schizophrenia: an NIMH workshop on definitions, assessment, and research opportunities. *Schizophr Bull*, *34*(6), 1211-1220. https://doi.org/10.1093/schbul/sbm145

Guhn, A., Merkel, L., Hubner, L., Dziobek, I., Sterzer, P., & Kohler, S. (2020). Understanding versus feeling the emotions of others: How persistent and recurrent depression affect empathy. *Journal of Psychiatric Research*, *130*, 120-127.

Hausberg, M. C., Schulz, H., Piegler, T., Happach, C. G., Klöpper, M., Brütt, A. L., Sammet, I., & Andreas, S. (2012). Is a self-rated instrument appropriate to assess mentalization in patients with mental disorders? Development and first validation of the mentalization questionnaire (MZQ). *Psychother Res*, *22*(6), 699-709. https://doi.org/10.1080/10503307.2012.709325

Jimenez, E., Sole, B., Arias, B., Mitjans, M., Varo, C., Reinares, M., Bonnin, C. D. M., Ruiz, V., Saiz, P. A., Garcia-Portilla, M. P., Buron, P., Bobes, J., Amann, B. L., Martinez-Aran, A., Torrent, C., Vieta, E., & Benabarre, A. (2017). Impact of childhood trauma on cognitive profile in bipolar disorder. *Bipolar Disord*, *19*(5), 363-374. https://doi.org/10.1111/bdi.12514

Kabat-Zinn, J. (2003). Mindfulness-based interventions in context: past, present, and future.

Kilian, S., Asmal, L., Chiliza, B., Olivier, M. R., Phahladira, L., Scheffler, F., Seedat, S., Marder, S. R., Green, M. F., & Emsley, R. (2018). Childhood adversity and cognitive function in schizophrenia spectrum disorders and healthy controls: evidence for an association between neglect and social cognition. *Psychological Medicine*, *48(13)*, 2186-2193.

Kincaid, D., Shannon, C., Boyd, A., Hanna, D., McNeill, O., Anderson, R., Francis-Naylor, M., & Mulholland, C. (2018). An investigation of associations between experience of childhood trauma and political violence and theory of mind impairments in schizophrenia. *Psychiatry Research*, *270*, 293-297.

Langdon, R., & Coltheart, M. (1999). Mentalising, schizotypy, and schizophrenia. *Cognition*, *71*(1), 43-71. https://doi.org/10.1016/s0010-0277(99)00018-9

Langdon, R., Coltheart, M., & Ward, P. B. (2006). Empathetic perspective-taking is impaired in schizophrenia: evidence from a study of emotion attribution and theory of mind. *Cogn Neuropsychiatry*, *11*(2), 133-155. https://doi.org/10.1080/13546800444000218

Langdon, R., Ward, P. B., & Coltheart, M. (2010). Reasoning anomalies associated with delusions in schizophrenia. *Schizophr Bull*, *36*(2), 321-330. https://doi.org/10.1093/schbul/sbn069

Lysaker, P., Clements, C., Plascak-Hallberg, C., Knipscheer, S., & Wright, D. (2002). Insight and Personal Narratives of Illness in Schizophrenia. *Psychiatry*, *65*, 197-206. https://doi.org/10.1521/psyc.65.3.197.20174

Lysaker Paul, H. P. H., Gumley, A., Brune, M., Vanheule, S., Buck, K. D., & Dimaggio, G. (2011). Deficits in the ability to recognize one's own affects and those of others: Associations with neurocognition, symptoms and sexual trauma among persons with schizophrenia spectrum disorders. *Consciousness and Cognition*, *20(4)*, 1183-1192.

Lysaker, P. H., Gumley, A., Luedtke, B., Buck, K. D., Ringer, J. M., Olesek, K., Kukla, M., Leonhardt, B. L., Popolo, R., & Dimaggio, G. (2013). Social cognition and metacognition in schizophrenia: evidence of their independence and linkage with outcomes. *Acta Psychiatrica Scandinavica*, *127*(3), 239-247. https://doi.org/https://doi.org/10.1111/acps.12012

Mansueto, G., Schruers, K., Cosci, F., van Os, J., Alizadeh, B. Z., Bartels-Velthuis, A. A., van Beveren, N. J., Bruggeman, R., Cahn, W., de Haan, L., Delespaul, P., Meijer, C. J., Myin-Germeys, I., Kahn, R. S., Schirmbeck, F., Simons, C. J. P., van Haren, N. E. M., & van Winkel, R. (2019). Childhood adversities and psychotic symptoms: The potential mediating or moderating role of neurocognition and social cognition. *Schizophrenia Research*, *206*, 183-193.

McDonald, S., Flanagan, S., Rollins, J., & Kinch, J. (2003). TASIT: A new clinical tool for assessing social perception after traumatic brain injury. *J Head Trauma Rehabil*, *18*(3), 219-238. https://doi.org/10.1097/00001199-200305000-00001

Mitchell, R. L., & Phillips, L. H. (2015). The overlapping relationship between emotion perception and theory of mind. *Neuropsychologia*, *70*, 1-10. https://doi.org/10.1016/j.neuropsychologia.2015.02.018

Nazarov, A., Frewen, P., Parlar, M., Oremus, C., MacQueen, G., McKinnon, M., & Lanius, R. (2014). Theory of mind performance in women with posttraumatic stress disorder related to childhood abuse. *Acta Psychiatr Scand*, *129*(3), 193-201. https://doi.org/10.1111/acps.12142

Page, M. J., McKenzie, J. E., Bossuyt, P. M., Boutron, I., Hoffmann, T. C., Mulrow, C. D., Shamseer, L., Tetzlaff, J. M., Akl, E. A., Brennan, S. E., Chou, R., Glanville, J., Grimshaw, J. M., Hróbjartsson, A., Lalu, M. M., Li, T., Loder, E. W., Mayo-Wilson, E., McDonald, S., . . . Moher, D. (2021). The PRISMA 2020 statement: an updated guideline for reporting systematic reviews. *BMJ*, *372*, n71. https://doi.org/10.1136/bmj.n71

Palmier-Claus, J., Berry, K., Darrell-Berry, H., Emsley, R., Parker, S., Drake, R., & Bucci, S. (2016). Childhood adversity and social functioning in psychosis: Exploring clinical and cognitive mediators. *Psychiatry Research*, *238*, 25-32.

Parlar, M., Frewen, P., Nazarov, A., Oremus, C., MacQueen, G., Lanius, R., & McKinnon, M. C. (2014). Alterations in empathic responding among women with posttraumatic stress disorder associated with childhood trauma. *Brain Behav*, *4*(3), 381-389. https://doi.org/10.1002/brb3.215

Petersen, R., Brakoulias, V., & Langdon, R. (2016). An experimental investigation of mentalization ability in borderline personality disorder. *Comprehensive Psychiatry*, *64*, 12-21.

Rnic, K., Sabbagh, M. A., Washburn, D., Bagby, R. M., Ravindran, A., Kennedy, J. L., Strauss, J., & Harkness, K. L. (2018). Childhood emotional abuse, physical abuse, and neglect are associated with theory of mind decoding accuracy in young adults with depression. *Psychiatry Research*, *268*, 501-507.

Rokita, K. I., Dauvermann, M. R., Mothersill, D., Holleran, L., Holland, J., Costello, L., Cullen, C., Kane, R., McKernan, D., Morris, D. W., Kelly, J., Gill, M., Corvin, A., Hallahan, B., McDonald, C., & Donohoe, G. (2021). Childhood trauma, parental bonding, and social cognition in patients with schizophrenia and healthy adults. *Journal of Clinical Psychology*, *77(1)*, 241-253.

Schalinski, I., Teicher, M. H., Carolus, A. M., & Rockstroh, B. (2018). Defining the impact of childhood adversities on cognitive deficits in psychosis: An exploratory analysis. *Schizophrenia Research*, *192*, 351-356.

Schreiter, S., Pijnenborg, G. H. M., & aan het Rot, M. (2013). Empathy in adults with clinical or subclinical depressive symptoms. *Journal of Affective Disorders*, *150*(1), 1-16. https://doi.org/https://doi.org/10.1016/j.jad.2013.03.009

Semerari, A., Carcione, A., Dimaggio, G., Falcone, M., Nicolò, G., Procacci, M., & Alleva, G. (2003). How to evaluate metacognitive functioning in psychotherapy? The metacognition assessment scale and its applications. *Clinical Psychology & Psychotherapy*, *10*, 238-261.

Sharmin, S., Kypri, K., Khanam, M., Wadolowski, M., Bruno, R., & Mattick, R. P. (2017). Parental supply of alcohol in childhood and risky drinking in adolescence: systematic review and meta-analysis. *International Journal of Environmental Research and Public Health*, *14*(3), 287.

Simon, M., Nemeth, N., Galber, M., Lakner, E., Csernela, E., Tenyi, T., & Czeh, B. (2019). Childhood Adversity Impairs Theory of Mind Abilities in Adult Patients With Major Depressive Disorder. *Frontiers in Psychiatry*, *10 (no pagination)*, Article 867.

Spreng, R. N., McKinnon, M. C., Mar, R. A., & Levine, B. (2009). The Toronto Empathy Questionnaire: scale development and initial validation of a factor-analytic solution to multiple empathy measures. *J Pers Assess*, *91*(1), 62-71. https://doi.org/10.1080/00223890802484381

Trauelsen, A. M., Gumley, A., Jansen, J. E., Pedersen, M. B., Nielsen, H. G. L., Haahr, U. H., & Simonsen, E. (2019). Does childhood trauma predict poorer metacognitive abilities in people with first-episode psychosis? *Psychiatry Research*, *273*, 163-170.

Vaskinn, A., Melle, I., Aas, M., & Berg, A. O. (2021). Sexual abuse and physical neglect in childhood are associated with affective theory of mind in adults with schizophrenia. *Schizophrenia Research: Cognition*, *23 (no pagination)*, Article 100189.

Weijers, J., Fonagy, P., Eurelings-Bontekoe, E., Termorshuizen, F., Viechtbauer, W., & Selten, J. P. (2018). Mentalizing impairment as a mediator between reported childhood abuse and outcome in nonaffective psychotic disorder. *Psychiatry Research*, *259*, 463-469.

Wells, A., & Cartwright-Hatton, S. (2004). A short form of the metacognitions questionnaire: properties of the MCQ-30. *Behaviour Research and Therapy*, *42*(4), 385-396. https://doi.org/https://doi.org/10.1016/S0005-7967(03)00147-5

World Health, O. (2004). ICD-10 : international statistical classification of diseases and related health problems : tenth revision. In (2nd ed.). Geneva: World Health Organization.
